# A *Myc*-regulated transcriptional network controls B-cell fate in response to BCR triggering

**DOI:** 10.1186/1471-2164-10-323

**Published:** 2009-07-17

**Authors:** Jernej Murn, Irena Mlinaric-Rascan, Pierre Vaigot, Olivier Alibert, Vincent Frouin, Xavier Gidrol

**Affiliations:** 1 CEA, DSV, IRCM, Laboratoire d'Exploration Fonctionnelle des Génomes, Evry 91057, France; 2 Faculty of Pharmacy, University of Ljubljana, 1000 Ljubljana, Slovenia; 3 Current address : Cancer Center, Cold Spring Harbor Laboratory, Cold Spring Harbor, NY 11724, USA; 4 Current address : CEA, DSV, IRTSV, Laboratoire Biopuces, 17 rue des Martyrs, 38054 Grenoble Cedex 09, France

## Abstract

**Background:**

The B cell antigen receptor (BCR) is a signaling complex that mediates the differentiation of stage-specific cell fate decisions in B lymphocytes. While several studies have shown differences in signal transduction components as being key to contrasting phenotypic outcomes, little is known about the differential BCR-triggered gene transcription downstream of the signaling cascades.

**Results:**

Here we define the transcriptional changes that underlie BCR-induced apoptosis and proliferation of immature and mature B cells, respectively. Comparative genome-wide expression profiling identified 24 genes that discriminated between the early responses of the two cell types to BCR stimulation. Using mice with a conditional *Myc*-deletion, we validated the microarray data by demonstrating that *Myc *is critical to promoting BCR-triggered B-cell proliferation. We further investigated the *Myc-*dependent molecular mechanisms and found that *Myc *promotes a BCR-dependent clonal expansion of mature B cells by inducing proliferation and inhibiting differentiation.

**Conclusion:**

This work provides the first comprehensive analysis of the early transcriptional events that lead to either deletion or clonal expansion of B cells upon antigen recognition, and demonstrates that *Myc *functions as the hub of a transcriptional network that control B-cell fate in the periphery.

## Background

The capacity of the mammalian immune system to discriminate between foreign chemical entities and the body's own components is critically dependent upon the correct choice between life and death of immune cells. While recognition of autoantigens by the B cell receptor (BCR) on self-reactive immature B cells leads to their deletion by apoptosis, foreign, antigen-specific, mature B lymphocytes respond to BCR engagement by clonal expansion [1]. Increasing evidence suggests that immature and mature B cells are differently adapted to signal transduction via BCR [2,3]. Among the potential mechanisms for regulating this functional dichotomy are differences in the partitioning of BCR signaling components to lipid rafts [4], the expression of B-cell co-receptors [5,6], basal levels of distal effector molecules (e.g, transcription factors and anti-apoptotic molecules) [7,8], and the levels of activation of specific kinases and phosphatases. Immature B cells do not proliferate in response to BCR triggering, but they do mount an abortive attempt to enter the cell cycle [9] and, similar to mature B cells, undergo a rapid unfolded protein response [10], which indicates an adaptation to an increased demand for protein folding. Recently, a model has emerged that suggests that B-cell fate is determined by the balance between survival and death signals initiated through the BCR [3].

Although both the BCR-induced death of immature B cells and the BCR-induced expansion of conventional mature B cells are critically dependent upon the *de novo *transcription and translation of genes [11-14], no study to date has investigated the mechanisms for the opposing phenotypes at the transcriptome level. To address this issue systematically, we undertook a genome-wide screen to identify the BCR-regulated transcriptional module and used functional studies to establish *Myc *as a hub of the network that shapes the response to BCR-priming.

## Methods

### Mice and antibodies

For transcriptional analysis of the BCR-triggered responses of T_1 _immature and mature B cells, male C57BL/6 mice were studied at 4 weeks (The Jackson Laboratory, Bar Harbor, Maine, USA). Mice with B lineage cell-specific conditional *Myc *inactivation were generated as described [15]. All experiments used background-, age- and sex-matched controls. All mutant mice and their controls were used at an age between 4 and 10 months. Affinity-purified anti-mouse CD16/32, FITC-conjugated anti-mouse B220, PE-conjugated anti-mouse CD23 and APC-conjugated anti-mouse CD93/C1qRp antibodies were purchased from eBioscience, (San Diego, California, USA). Goat F(ab')_2 _anti-mouse IgM, μ-chain specific, was purchased from Jackson ImmunoResearch (West Grove, Pennsylvania, USA).

### B cell purification, flow cytometry and cell culture

Single-cell suspensions were prepared from isolated spleen. Untouched B cells were recovered by depletion of non-B cells using the B Cell Isolation Kit (Miltenyi Biotec, Bergisch Gladbach, Germany). The purity of B cells, which was measured by the percentage of B220^+ ^cells, was more than 95%. Cells were either used as the total B cell fraction or were pre-incubated with purified anti-CD16/32 to block Fc receptors and further sorted as CD93^+^CD23^- ^(T1 immature) and CD93^-^CD23^+ ^(mature) by fluorescence activated cell sorting (MoFlo, Cytomation, Glostrup, Denmark), as previously described [16,17]. B cells were cultured at a density of 2 × 10^6 ^cells/ml in IMDM medium, which was supplemented with 5% FCS, 100 U/ml of penicillin, 100 μg/ml of streptomycin, 2 mM glutamine and 50 μM β-mercaptoethanol, in a humidified incubator at 37°C and 5% CO_2_. The cells were stimulated with or without anti-μ IgM F(ab')_2 _at 30 μg/ml.

### Phenotypic analyses

The proliferation of B cells was assessed by determining their ATP content using a bioluminescent assay (ViaLight Plus, Cambrex, East Rutherford, New Jersey, USA). Tests were performed in 96-well plates at 1 × 10^5 ^cells/well according to the manufacturer's instructions. Cell viability was monitored by the exclusion of 0.4% trypan blue solution and visualized by light microscopy. The extent of apoptosis was assessed by measuring the activities of caspases 3 and 7 using the Apo-ONE Homogeneous Caspase-3/7 Assay, according to the manufacturer's instructions (Promega, Charbonnieres, France). The cell cycle was analyzed as previously described [18]. Phase-contrast microscopy was used to photograph isolated B cells in culture after 48 h of treatment (Olympus 1X70, Olympus, Tokyo, Japan). The levels of secreted IgM in sample supernatants were determined using Mouse IgM ELISA Quantitation Kit (Bethyl Laboratories, Montgomery, Texas, USA).

### Microarrays, hybridization and data analysis

DNA arrays were prepared in house from the NIA Mouse 15K cDNA clone set [19]. Total RNA from sorted T_1 _immature and mature B cells of 10 mice was extracted using the Trizol reagent (Invitrogen, Cergy Pontoise, France), followed by one round of amplification with the MessageAmp™ Kit (Ambion ABI, Austin, Texas, USA). The integrity of the RNA samples was verified using agarose gel electrophoresis and the Bioanalyser 2100 (Agilent, Massy, France). Amplified RNA was reverse-transcribed using random primers, and aminoallyl-dUTP was incorporated for indirect labeling with Cy3 and Cy5. Each labeled cDNA sample was hybridized to the arrays at least six times using a dye-swap strategy. All samples were hybridized against amplified Universal Mouse Reference RNA (Stratagene, La Jolla, California, USA). Arrays were scanned with the ScanArray5000 (Packard Instruments, Rungis, France) or Genepix 4000B scanner, and feature extraction was performed with the Genepix Pro 4.0 software (Axon Instruments MDC, Sunnyvale, California, USA). Data analysis, including intensity-dependent Lowess normalization of the raw data, was done with GeneSpring 7.0 software (Agilent, Massy, France). The data are available in the Gene Expression Omnibus (GEO; accession number GSE9215). To analyze gene promoters, we used the web-based Genomatix software (Genomatix, Ann Harbor, Michigan, USA; ), which is a bioinformatics tool that utilizes a large library of matrix descriptions for known transcription factor binding sites and algorithms to locate matches in DNA sequences. To study how BCR-recruited molecules and their interactions may determine the phenotypic outcome of B cells, Ingenuity Pathway Analysis (IPA, Rewood City, California, USA; ) [20], which is a knowledge-based software package, was used to identify the putative BCR-regulated functional module.

### Quantitative real-time PCR and network analysis

qPCR was performed on total RNA from two independent biological replicates. Samples were processed on an ABI PRISM 7700 Sequence Detection System (Applied Biosystems, Austin, Texas, USA) using the SYBR Green JumpStart Taq ReadyMix (Sigma-Aldrich, Lyon, France). All qPCR data were generated using *Hprt1 *as a reference (house-keeping) gene. This gene was chosen based on the results of the microarray analysis, which showed no significant variation in the expression of *Hprt1 *among any of the samples tested. We also verified our qPCR data by performing calculations using a second house-keeping gene, *Cox1*, and obtained similar results. Primer sequences are available upon request.

## Results

### Opposite responses of immature and mature B cells to BCR triggering

**Figure 1:**
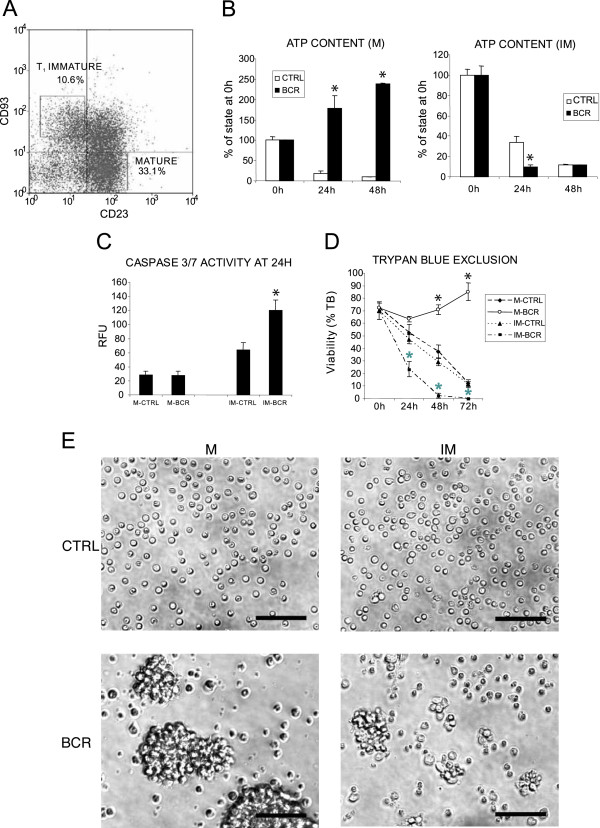
**Opposite responses of T1 immature and mature B cells to BCR triggering**. **(A) **Fluorescence activated cell sorting (FACS) of magnetically separated, untouched B lymphocytes from the spleens of C57BL/6 mice using APC-conjugated CD93-specific and PE-conjugated CD23-specific antibodies. T1 immature (CD93^+^, CD23^-^) and mature (CD93^-^, CD23^+^) B cells were sorted, as shown by the two rectangles. **(B-E) **Proliferation and apoptosis of sorted mature (M) and T1 immature (IM) B cells in response to BCR stimulation. Cells were incubated *in vitro *with (BCR) or without (ctrl) anti-μ F(ab')_2 _for the indicated periods of time and assayed for **(B) **ATP content, **(C) **caspase 3/7 activity and **(D) **trypan blue (TB) exclusion. Values are mean ± s.e.m. of three independent experiments. *, *P *< 0.01, compared to the respective control samples using Student's *t*-test (black and grey asterisks in **(D) **refer to the comparison of mature and immature samples, respectively). RFU, relative fluorescence units. **(E) **Phase contrast microscopy of mature and T1 immature B cells after 48 h of stimulation with anti-μF(ab')_2 _in culture. Lower panels show BCR-triggered large clusters of proliferating mature cells (left) and smaller groups of mainly apoptotic T1 immature B cells (right). Scale bar, 50 μm. Non-B cells from spleen did not respond to the treatment with anti-μ IgM antibodies, as determined by these assays (data not shown).

To understand the transcriptional basis for the BCR-regulated sensitivity to the apoptosis of immature B cells versus the proliferative response of mature B cells, we used antibodies to surface IgM to mimic antigen binding by cross-linking the BCR on both cell types *in vitro*. To assure minimal developmental differences between both cell populations, we chose to use freshly isolated splenic transitional immature B cells, as they represent the latest stage of immature B cells and reside in a microenvironment that is closest to the mature follicular compartment. After negative selection of B cells, we used surface expression of CD93 and CD23 as selection markers to sort cells, i.e. the transitional 1 (T1) immature cells were CD93^+^CD23^- ^and the mature cells were CD93^-^CD23^+ ^(Fig. 1A) [16,17]. Since these markers do not interact with the BCR, this selection strategy allowed us to avoid uncontrolled triggering of the receptor during sorting. In accordance with previous observations, cross-linking of BCR on freshly isolated cells induced apoptosis of the T1 immature cells and a mitogenic response of the mature B cells (Fig. 1B–E) [16]. The effects of BCR-stimulation on the sorted B cell populations were assayed by ATP content measurements to evaluate the proliferation status of cells, caspase 3/7 activity to measure the extent of apoptosis and trypan blue exclusion to estimate the viability of cells in culture. In addition, phase contrast microscopy showing large clusters of proliferating mature B cells and much smaller clumps of apoptotic immature cells upon BCR stimulation confirmed the results of the above assays. These observations prompted us to determine how as little as the one day of differentiation that separates the two B cell populations in the spleen results in the switch of the BCR-mediated response from death to intense proliferation.

### Changes in gene expression underlying BCR-induced apoptosis and proliferation

We analyzed transcriptional changes in response to BCR ligation using cDNA microarrays containing 15,247 unique oligo(dT)-primed cDNA clones, which were primarily derived from early embryonic cDNA libraries [19]. All samples were hybridized against commercially available "universal" RNA, which allowed us to compare any samples within our experimental design (Additional file 1A). By comparing the gene expression profiles of mature versus T1 immature B lymphocytes, 44 genes (excluding EST and RIKEN sequences) that differed significantly between both differentiation stages were identified. The observed changes in gene expression largely conformed to observations from other cell types or agreed with their known functions in B cell development, notably *CD24a*, *Fcgr2b*, *Id2*, *Myc *and *Prkcd *(Table 1 and Additional file 2). Interestingly, 82% of these genes were down-regulated in mature B cells, and the majority has been described to act against differentiation and proliferation or to favor apoptosis induction.

**Table 1:** Differentially regulated genes between mature and T_1 _immature B cells.

Gene symbol	Function	M/IM
*Syngr2*	B-cell and neuronal differentiation	2.1
*Idi1*	cholesterol synthesis	1.7
*Myc*	B-cell proliferation, differentiation	1.7
*Litaf*	regulates inflammatory cytokine expression	1.5
*Spag9*	positively regulates MAPK signaling pathways	1.4
*Rnf5*	inhibits cell motility	1.4
*Mfn2*	mitochondrial fusion	1.3
*Txnip*	immune cell differentiation	1.3
*Mfap3*	no data	-1.3
*Bnip3l*	apoptosis	-1.3
*Clta*	intracellular protein transport	-1.5
*Actb*	cytoskeleton structure	-1.5
*Pik3c2a*	survival and proliferation	-1.6
*Acad9*	mitochondrial fatty acid oxidation	-1.6
*Prkcd*	inhibits B-cell proliferation and differentiation	-1.6
*Vezf1*	angiogenesis	-1.6
*Pcyt1a*	apoptosis	-1.7
*Cecr2*	chromatin remodeling	-1.7
*Zcchc17*	no data	-1.7
*Coro1b*	neurite outgrowth	-1.7
*Atp5c1*	no data	-1.8
*H2afz*	embryonic development	-1.8
*Cd24a*	B-cell apoptosis; development of immature B cells	-2.1
*Npc2*	cholesterol transport	-2.1
*Emb*	cell adhesion during development	-2.2
*Fcgr2b*	inhibits B-cell proliferation	-2.2
*Emx2*	neuronal growth and development	-2.3
*Cst3*	reduces antigen presenting capacity of dendritic cells	-2.3
*Id2*	inhibits B-cell differentiation	-2.6
*Cul7*	regulates B-cell differentiation	-2.6
*Wwp1*	T-cell differentiation; embryogenesis	-2.7
*Jun*	apoptosis, proliferation, differentiation	-2.7
*Ctsb*	apoptosis	-2.8
*Ifitm3*	inhibits proliferation	-3.1
*Nr2f2*	regulates organogenesis	-3.1
*Atp1b1*	inhibits T-cell activation	-3.2
*Slc40a1*	iron export	-3.6
*Phtf*	regulates spermatogenesis	-3.7
*Hoxb3*	regeneration of stem cells	-3.8
*C1qa*	reduces T cell-dependent immune response	-5.1
*Laptm5*	B-cell differentiation; inhibits maturation of dendritic cells	-6.2
*Hmox1*	inhibits B-cell activation; inhibits T-cell proliferation	-7.4
*Hba-a1*	oxygen transport	-7.9
*Lyzs*	defense response to bacteria	-23.8

Genes discriminating transcriptional profiles of splenic mature and T_1 _immature B lymphocytes. The analysis was based on one-way ANOVA (P < 0.01) of means of at least five replicate microarray experiments. Genes are ranked by the ratio of values for mature (M) versus immature (IM) B cells. For those genes with described functions, either their B-cell associated functions or, if unknown, primary functions in other cell types are listed. Differentially expressed EST and RIKEN sequences (46 in total) are not shown but can be recovered from the GEO database (GSE9215).

We next applied a hierarchical clustering analysis of gene expression patterns over time to assess the relative similarities between immature and mature B cells in response to BCR engagement. We observed a clear segregation of anti-IgM treated samples from untreated controls, with the latter being further separated by the stage of differentiation. In contrast, treated samples were grouped according to the time of incubation (Additional file 1B).

A similarity between the responses of immature and mature cells to BCR cross-linking was also evident when we examined the number of differentially expressed genes relative to the zero hour time point. Approximately equivalent numbers of genes were induced and repressed in both cell populations at incubation time of 2 and 8 h. In addition, treatment with anti-IgM influenced the expression of at least 3 times more genes than medium alone at each time point (Additional file 1C). However, a general analysis of gene functions following BCR triggering at 2 h revealed a substantial difference in the early transcriptional responses of immature and mature B cells. As opposed to immature cells, BCR triggering on mature B cells elicited a strong transcriptional perturbation of genes that regulated biosynthesis, which indicated an adaptation for the upcoming proliferative burst (Additional file 3). A much less pronounced induction of cell growth-promoting genes in stimulated immature B cells likely reflects their abortive attempt to proliferate, as previously described [9,10]. Taken together, these data demonstrate that, although the immature compartment exhibits a gene expression pattern that is distinct from that of mature B cells, both cell populations respond to BCR triggering with a relatively similar transcriptional program.

To identify the early genes that discriminate the responses of the two cell types to BCR stimulation, we used two-way ANOVA (*P *< 0.05) at the two-hour time point, while taking maturity stage and treatment as two independent variables. This approach allowed us to identify BCR-induced differences in gene expression with high sensitivity and to discriminate them from any transcriptional variations due to the developmental stage of the cells. The analysis identified 24 genes that likely determined the phenotypic outcome of the mature and immature cells upon BCR triggering. As expected, candidate genes from the top of the list in Table 2 have been shown to stimulate cell growth and proliferation, whereas genes from the bottom have been associated with negative signaling. To validate the array data, we confirmed the expression of 23 out of the 24 genes by quantitative real-time PCR (qPCR) on total RNA isolated from two independent biological replicates (Table 2 and Additional file 4).

**Table 2:** BCR-regulated genes discriminating immature and mature B cells at 2 h.

		microarrays			qPCR	
			
		BCR/ctrl			BCR/ctrl	
				
Gene symbol	Function	IM	M	M/IM	IM	M
*Ptger4*	B-cell activation and differentiation	1.7	4.6	**2.8**	1.8	4.8
*Marcksl1*	proliferation, cell adhesion, neurosecretion	2.6	6.1	**2.4**	2.2	4.3
*Myc*	B-cell proliferation, differentiation, apoptosis	3.8	8.9	**2.3**	4.1	12.9
*Crsp9*	proliferation	1.4	3.0	**2.1**	1.7	3.1
*Chchd4*	no data	1.6	3.3	**2.1**	1.7	3.1
*Wdr55*	rRNA synthesis, cell cycle progression	1.3	2.4	**1.9**	1.7	1.9
*Ifrd1*	differentiation, proliferation	1.3	2.2	**1.7**	1.4	2.3
*Eif3s1*	T-cell activation	1.3	2.3	**1.7**	1.7	2.0
*Idi1*	cholesterol synthesis	1.6	2.7	**1.7**	1.6	2.3
*M6pr*	lysosomal enzyme transport	1.4	2.5	**1.7**	1.1	2.0
*Nola2*	T-cell activation	1.4	2.3	**1.7**	1.7	2.2
*Pdhx*	electron transport	1.8	3.0	**1.7**	-1.3	-1.4
*Hrb*	spermiogenesis	1.5	2.3	**1.5**	1.1	2.5
*Lyar*	cell growth	1.3	1.9	**1.5**	1.2	2.7
*Irx3*	heart and CNS development	-1.0	1.4	**1.4**	-1.2	1.5
*Rpo1-3*	cell growth	1.1	1.5	**1.4**	1.1	1.5
*Ptdss1*	cell growth	-1.0	1.4	**1.4**	-1.4	1.4
*Stt3b*	oligosaccharyltransferase activity	1.9	1.3	**-1.5**	2.6	-1.3
*Plekha2*	B-cell receptor signaling	-1.7	-2.6	**-1.6**	-2.5	-5.4
*Fbxo22*	no data	1.0	-1.6	**-1.6**	-1.4	-2.0
*Jun*	apoptosis, proliferation, differentiation	1.1	-1.6	**-1.8**	-1.1	-3.4
*3110001A13Rik*	no data	-1.7	-3.6	**-2.1**	-1.6	-5.1
*Spata13*	no data	-1.8	-4.8	**-2.7**	-2.5	-16.7
*Tmem23*	apoptosis	-1.9	-5.3	**-2.8**	-2.8	-20.0

Differentially expressed genes in immature (IM) and mature (M) B cells at 2 h of BCR stimulation identified by two-way ANOVA (*P *< 0.05). Expression changes are shown as ratios of BCR triggered (BCR) versus control (ctrl) B cells at 2 h. Data are means of at least five replicate microarray experiments and at least two independent triplicate RT-PCR results.

### Genes co-regulated by BCR signaling share several cis-acting sequences in their promoter regions

**Figure 2:**
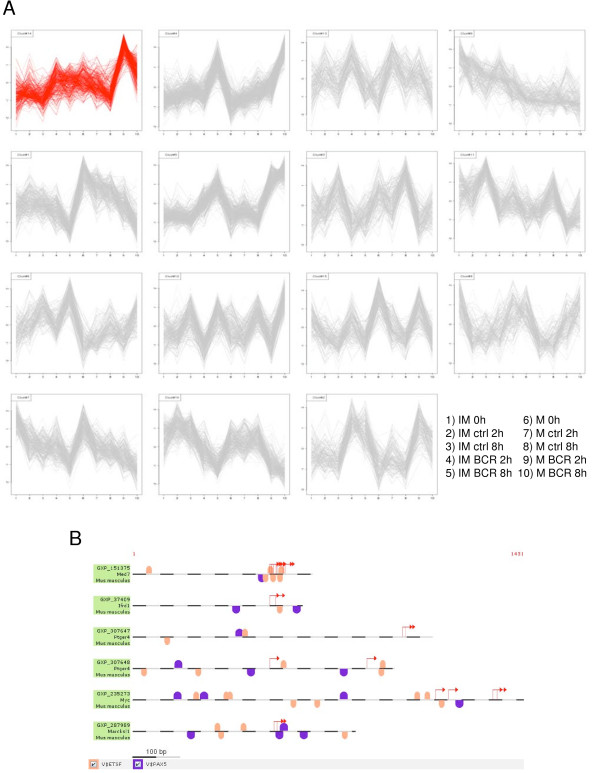
**Gene expression profiles of T1 immature and mature B lymphocytes in response to BCR triggering**. **(A) ***k*-means clustering using standard correlations was applied at several *k *values to classify all 2,454 genes (represented by 3,505 probes on the array) that showed differential expression between any sets of samples (see the key on the right). As shown in Additional file 5A, the result is independent of the selected *k *value. Shown are the clusters generated by running the analysis at k = 15. The cluster highlighted in red consists of *Ptger4*, *Marcksl1*, *Myc*, *Crsp9*, *Ifrd1 *and *Pdhx*. Data are means of normalized values of at least five microarray experiments. Note that the data from two different probes each (a, b) for *Marcksl1 *and *Myc *were used for clustering. **(B) **Transcription factor (TF) binding sites that are common to six promoter regions from the selected cluster of genes. A graphical representation of the matches shows the location for each binding site recognized by either the ETS family of TFs or *Pax5 *relative to the predicted transcription initiation sites (red arrows). As *Pdhx *was not validated by qPCR, it was excluded from the analysis. Note that we included two known promoter regions for *Ptger4*. *Crsp9 *is also known as *Med7*. The analysis was performed using the Genomatix software . Only antibody producing cell-specific binding sites were analyzed. The statistics regarding the occurrence of these binding sites are provided in Additional file 5B.

Among the 24 genes, several of the most highly up-regulated were co-clustered by *k-*means analysis (Fig. 2A & Additional file 5A). Note that one of the clusters contained five genes that had very similar expression profiles across all the conditions. These five genes *Crsp9, Ifrd1*, *Myc*, *Marcksl1 *and *Ptger4*, were also identified as the key cell fate-regulators (Table 2). Assuming that co-expressed genes are regulated by the same transcription factors and share common binding sites in their upstream regions on the one hand and that they should respond to BCR signaling on the other hand, we screened the promoter regions of differentially expressed genes at 2 h for common regulatory sequences. For this purpose, a web-based MatInspector software from Genomatix was used to search promoter regions of the five co-clustered genes for known transcription factor binding sites. Interestingly, all five genes shared two types of antibody producing cell-specific binding sites (Fig. 2B) One included those that were recognized by the ETS family of transcription factors, such as *Elk1*, which is known to respond to BCR signaling [21], and the other included binding sites for *Pax5*, which is a key transcription factor that controls B-cell proliferation and differentiation and which has also been linked to BCR stimulation [22,23]. We then tested for the statistical significance of the occurrence of these binding sites compared either to the entire genome or the promoters of all genes. The results presented in Additional file 5B show that *Pax5*, but not the ETS family members' binding sites, are overrepresented in the five genes of interest. These data indicate the putative binding sites and the identity of transcription factors that may be responsible for the BCR-mediated co-regulation of the most critical cell-fate decision-making genes, and it is intriguing that these are the only predicted B cell-specific transcription factor binding sites that are found in the promoters of all five genes.

### *Myc *promotes BCR-dependent clonal expansion of mature B cells by inducing proliferation and inhibiting their differentiation

**Figure 3:**
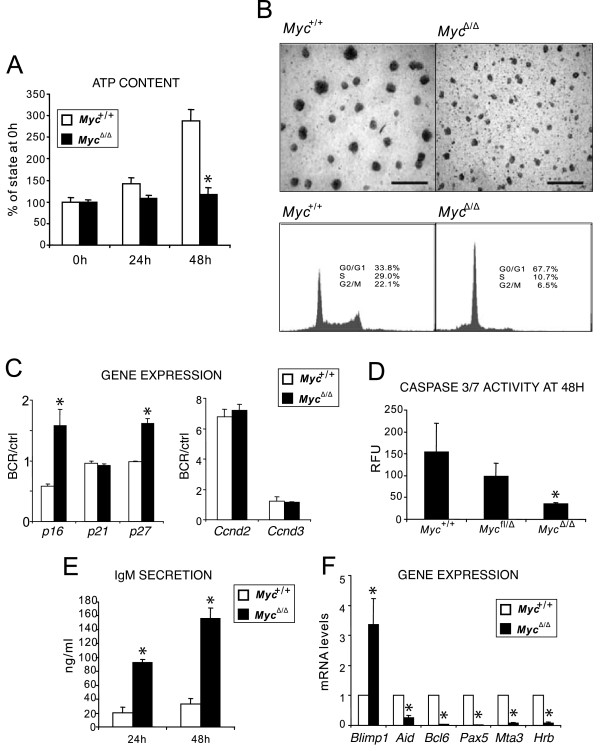
***Myc *up-regulation is required for BCR-triggered proliferation and differentiation arrest of B cells**. **(A-F) **These experiments were performed on magnetically separated total untouched B cells from spleen of transgenic mice and their wild-type controls. Cells were analyzed either freshly isolated or after *in vitro *incubation with (BCR) or without (ctrl) anti-μ F(ab')_2 _for the indicated periods of time. **(A) **Measurements of ATP content in cells that were treated with anti-μ F(ab')_2_. Results are mean ± s.d. of two independent experiments performed in triplicate. **(B) **Cells were incubated with anti-μ F(ab')_2 _unperturbed for 48 h, photographed under phase-contrast microscope (top) and then stained with propidium iodide (PI) for cell cycle analysis (bottom). Live cell gating is shown. Data are representative of two or three experiments. **(C) **Transcriptional regulation of cell cycling genes in mature *Myc*^+/+ ^and *Myc*^Δ/Δ ^B cells was examined by qPCR at 18 h of BCR-triggering. **(D) **Measurements of caspase 3/7 activity of cells treated with anti-μ F(ab')_2_. **(E) **ELISA of IgM in supernatants of unstimulated *Myc*^fl/Δ ^and *Myc*^Δ/Δ ^B cells. Data are mean ± s.e.m. of three independent experiments. **(F) **Steady-state mRNA levels of terminal differentiation-associated genes in resting mature *Myc*^+/+ ^and *Myc*^Δ/Δ ^B cells.

To examine whether the BCR-mediated up-regulation of *Myc *was the cause for the BCR-dependent clonal expansion of the B cells rather than a consequence of it, we used *Myc*^fl/fl^; *CD19*^cre^; *egfp *(fl/fl) mice, which show a B lineage cell-specific conditional *Myc *deletion in 60–70% of their splenic B cells [15]. Stimulation of BCR on *Myc*-deficient B cells resulted in a severely reduced proliferative response (Fig. 3A), yet these cells resisted apoptosis (Fig. 3D) and instead accumulated in the G0/G1 phase (Fig. 3B). Since *Myc *has been shown to exert an essential role in B-cell homeostasis [24], we further analyzed how *Myc *may control B-cell fate in response to BCR triggering. A survey of cell cycle-regulating genes indicated that this defect in BCR-triggered proliferation was mediated primarily by the up-regulation of cyclin-dependent kinase inhibitors p16 and p27 (Fig. 3C). Interestingly, when we incubated *Myc*^Δ/Δ ^cells in medium alone, we observed 3- to 5-fold increase in antibody secretion relative to *Myc*^fl/Δ ^or wild-type B cells, which indicated that the *Myc *deletion facilitated terminal differentiation to antibody-secreting plasma cells *in vivo *(Fig. 3E). Indeed, *Myc*^Δ/Δ ^B cells exhibited a terminally differentiated, plasma cell-like genotype that was characterized by the mandatory up-regulation of *Blimp-1 *and a near extinguishment of *Aid*, *Bcl6*, *Mta3 *and *Pax5 *expression, which are all known inhibitors of terminal B-cell differentiation (Fig. 3F). These data indicate that *Myc *up-regulation is critically important and causative for both the mitogenic responsiveness and the differentiation arrest upon BCR triggering.

### *Myc *functions as a hub gene of the BCR-operated transcriptional network to control life and death of B cells

**Figure 4:**
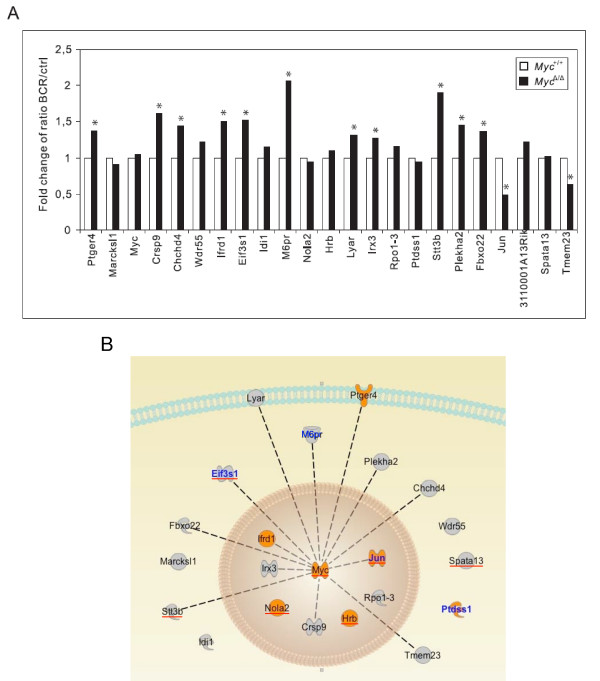
***Myc *functions as a hub gene of the BCR-operated transcriptional network to control life and death of B cells**. **(A) **Steady-state mRNA levels of genes selected by two-way ANOVA (Table 2) in mature *Myc*^+/+ ^and *Myc*^Δ/Δ ^B cells treated with (BCR) or without (ctrl) anti-μ F(ab')_2_. Each presented value is a normalized ratio of the expression level of each gene in BCR versus control sample for each genotype, i.e., *Myc*^+/+ ^and *Myc*^Δ/Δ^. The data were normalized by dividing each ratio value by the wild-type BCR/ctrl ratio value for the corresponding gene. *, *P *< 0.05 **(B) ***Myc*-regulated transcriptional network controlling B-cell fate in response to BCR triggering. The candidate decision-making genes that were identified by the transcriptome analysis (Table 2) were associated with *Myc *according to the data (significance) presented in panel **A**. Genes were connected by dashed lines to indicate our indirect evidence for their interactions. Several genes (*Wdr55, Spata13, Ptdss1, Rpo1-3, Hrb, Nola2, Idi1 *and *Ifrd1*) were not connected to *Myc *because we did not observe any change in BCR/ctrl ratios when comparing both genotypes, i.e., *Myc*^+/+ ^and *Myc*^Δ/Δ^. Genes in orange have been reported as neighbors of the *Myc*-network in B cells [37], and genes with a red underscore or genes whose names are written in blue have been described as direct *Myc *targets in [38] and [39], respectively.

Since we found *Myc *to have the most profound effect on BCR-recruited proliferation (Fig. 3A &3B) and know that *Myc *is one of the key genes that regulates B-cell functions, we investigated whether or not *Myc *affects the expression of other genes in our list (Table 2) in order to orchestrate the initial phase of the proliferative burst. For that purpose, we performed qPCR analysis of the early genes that were identified by two-way ANOVA upon BCR triggering of follicular mature *Myc*^Δ/Δ ^B cells, which still retain ~30%–40% of the wild-type level of *Myc *transcripts [15]. Interestingly, in comparison to wild-type controls, most genes were found to be significantly affected by the reduced expression of *Myc *(Fig. 4A). Of note, genes with a positive mature versus immature ratio, many of which have previously been demonstrated to favor proliferation (Table 2), showed either increased or unaltered expression upon BCR-priming. This is a likely result of an attempt to compensate for the lack of *Myc*-driven mitogenic signaling. In contrast, both known apoptosis-promoting genes, *Jun *and *Tmem23 *[25,26], were down-regulated, in accord with the observations that *Myc*-deficient B cells are resistant to apoptosis triggering. The induction of other genes with a negative mature versus immature ratio seems less clear; however, their up-regulation may be responsible for the cell cycle inhibition and G0/G1 arrest that follows BCR-triggering in *Myc*-deficient B cells. Thus, *Myc *acts as a hub of this network by directly or indirectly coordinating transcriptional activity of both stimulatory and inhibitory early response genes to control BCR-signaled cell fate. Fig. 4B summarizes our findings by depicting the identified *Myc*-regulated transcriptional network in which the candidate decision-making genes (Table 2) have been associated with *Myc*, based upon the results presented in Fig. 4A. It is noteworthy that most genes in this network, which mechanistically relates to the switch of the BCR-mediated response from death to intense proliferation of B cells in the periphery, have never been described as potential *Myc *targets.

## Discussion

The development of the correct repertoire of antigen specificity requires maturation of only those immune cells that will be able to specifically recognize the invading pathogens, but that stay inert towards all components of the host organism. In discriminating self from non-self, the mammalian immune system relies primarily on B cells, which undergo a step-by-step maturation process to attain immune competence [1,3]. Up until the last moments prior to reaching the mature stage, transitional immature B cells still undergo deletion by apoptosis upon antigen recognition [17]. It is thus intriguing to observe that the same signal can engage such contrasting cell fates in two closely related cell types and that the same phenomenon can be observed *in vitro *where culture conditions are also kept the same. Here, we examined the underlying mechanism that leads to these distinct biological responses that are central to the proper functioning of the adaptive immune system.

Regardless of the relatively solid evidence for the differential signal transduction pathways from the BCR, it has been demonstrated that both immature and mature B cell-responses are dependent upon *de novo *expression of genes [11-14]. However, to the best of our knowledge, only the study by Schories *et al*. investigated BCR-triggered global transcriptional changes that lead to a defined phenotype [14]. This work focused solely on the anti-IgM-induced apoptosis of an immature B-cell line, which makes the interpretation of the results difficult for two reasons. First, the "immortality" of a cell line significantly changes the initial phenotype from a short-lived and death-sensitive primary B cell to a robust and wildly proliferating "lymphoma" cell that exhibits markedly delayed kinetics of BCR-mediated death. Second, this study neglected the fact that BCR signaling can also induce the proliferation of B cells and that minute differences in gene expression may divert the BCR response from apoptosis to proliferation. Several other genome-wide transcriptional analyses of B-cell responses to BCR stimulation have been reported [27-30], but none of these studies considered comparing the opposing phenotypes, which proved to be critically important for the identification of the decision-making genes. For example, these studies have already identified *Ptger4*, *Marcksl1*, *Myc*, *Ifrd1 *and *Jun *among the top most differentially expressed genes, but they failed to discriminate them from other significantly regulated genes when their contribution to the studied B-cell phenotype was assigned.

Therefore, the identification of the critical molecules required a more structured approach to the complex transcriptional programs that are triggered by the BCR. We performed a comparative gene expression profiling of the opposing responses of immature and mature primary B cells to BCR cross-linking, which led us to identify 24 genes that could discriminate the early responses of the two cell types. Two-way ANOVA allowed us to disregard any transcriptional variations that were due to the developmental stage of the cells and to eliminate the genes showing the equivalent regulation by the BCR in immature and mature cells.

Interestingly, although the identified genes have not been mechanistically associated with BCR signaling before, with the exception of *Myc *and *Plekha2*, their functions described in other cell types largely agreed with the observed changes in expression. Several of the up-regulated genes in mature versus immature B cells have previously been shown to promote proliferation or differentiation in various cells, while *Myc *and *Ptger4 *have also been implicated in the development and homeostasis of B lymphocytes [18,31]. In contrast, limited data exist regarding the down-modulated genes. The pro-apoptotic functions of *Tmem23 *and *Jun *have been demonstrated, and *Plekha2 *is speculated to propagate negative signaling in B cells upon BCR ligation [25,26,32]. These data, together with the qPCR validation, suggest that the identified genes function to control the phenotypic outcome that is signaled by the BCR.

Previous studies have shown that BCR triggering up-regulates *Myc *expression in immature B cells at several differentiation stages, as well as mature B cells [9,33,34]. They have also suggested that *Myc *responsiveness to BCR ligation is developmentally regulated [35]. However, these studies focused mainly on the relationship between *Myc *expression and the negative selection of B cells by apoptosis. Here, we describe *Myc *as a direct regulator of both proliferation and apoptosis upon acute BCR signaling, especially since B cells lacking *Myc *respond very poorly to BCR ligation and are resistant to apoptosis. This is in agreement with a previous study that found 2- to 3-fold reduced numbers of total splenic *Myc*-deficient B cells and an impaired mitogenic response of these cells to stimulation with IL-4 and the anti-CD40 antibody [18]. Although *Myc *has long been appreciated as an inhibitor of differentiation in B cells [24,36], we showed here, for the first time, that the absence of *Myc *alone facilitates their terminal differentiation to antibody-secreting plasma cells.

*Myc *has recently been described as a "hub" gene that controls several cellular processes in many cell types, including B cells, by regulating up to 10 – 15% of all genes [37,38]. This vastness of the Myc transcriptional network makes it difficult to disentangle the contribution of Myc to any given cellular phenotype. However, focusing our study on the early transcriptional events of a specific biological process that occurs under defined *in vitro *conditions allowed us to identify a few changes in gene expression, including that of *Myc*, that discriminate between the two opposing cell fates. An independent experiment performed in a *Myc*-deficient background revealed that most of the identified genes (62%; Fig. 4A) are regulated by Myc. This extent of transcriptional control thus far exceeds the estimated 10 – 15% of all genes that are thought to be regulated by *Myc *and classifies *Myc *as the hub gene of our network. Among the 21 genes of the network, excluding the RIKEN sequence and *Myc *itself, 9 genes (43%; *Hrb*, *Ifrd*1, *Jun, Nola2*, *Ptdss1*, *Ptger4, Spata13, Stt3b *and *Eif3s1*) were described as part of a *Myc*-network in B cells [37,38], and an additional 2 genes (9.5%; *M6pr *and *Ptdss1*) have been annotated in the *Myc*-database [39]. The remaining 10 genes have never been reported as either direct or indirect targets of Myc. One of the most notable aspects of this network is the link between *Myc *and *Jun*, which seems to play a key role in the regulation of primary B cell-fate in response to BCR engagement. Indeed, ChIP-on-chip data analysis supports *Jun *as a direct target of *Myc *[40], and as we show here, *Myc *controls the BCR-dependent regulation of *Jun*.

## Conclusion

Our data identify the early changes in the transcriptional mechanisms that discriminate between *in vitro *mimicked negative selection by deletion and clonal expansion of B lymphocytes in spleen. The approach we have taken in this study provided a means to identify the decision-making genes in an otherwise complex transcriptional program that is triggered by the BCR. By integrating our data with previous knowledge, we identify a BCR-operated transcriptional network in which *Myc *functions as a master regulator of the growth and differentiation of B cells. Moreover, we showed that a reduced expression of *Myc*, as the most highly connected hub gene, results in a gross phenotypic change and the transcriptional perturbation of several genes in its neighborhood, thus justifying the role of *Myc *as a critical regulator of BCR-triggered life-or-death decisions. Since the wrong choice between life and death of B cells often leads to pathological conditions, such as immunodeficiency, autoimmunity or cancer, a thorough functional analysis of the molecules of the BCR-operated network may help in the design of better therapies for such diseases.

## Authors' contributions

JM, IMR and XG designed the research; JM and PV performed the experiments; JM, VF, OA and XG analyzed the data; and JM, IMR and XG drafted the paper.

## Supplementary Material

Additional file 1 **Changes in gene expression underlying BCR-induced apoptosis and proliferation**. **(A) **Experimental design used to identify the genes discriminating BCR-triggered transcriptional responses of immature and mature B cells at 2 and 8 h. Expression profiles used with two-way ANOVA are circled in red. Genes that passed the *P *< 0.05 criterion for each of the three parameters, which are maturity (M or IM), treatment (ctrl or BCR) and interaction between maturity and treatment, were identified as the discriminating genes for each time point (Table 1). **(B) **Unsupervised hierarchical cluster analysis of immature (IM) and mature (M) B cells of C57BL/6 mice both freshly isolated (0 h) and stimulated with (BCR) or without (ctrl) anti-F(ab')_2 _for 2 and 8 h. A hierarchical clustering algorithm based on standard correlations was applied in order to group all interrogated genes (13,043 gene features) on the basis of similarity in the pattern over all samples and all samples on the basis of similarity in the pattern over all genes. The data are presented in a matrix format, in which a column represents an individual sample and a row represents an individual gene. The red and blue colors in cells reflect high and low expression levels, respectively, as indicated in the scale bar (log-transformed scale). **(C) **Numbers of differentially expressed genes features in anti-IgM treated immature and mature B cells at 2 and 8 h versus the respective zero hour states (one-way ANOVA; *P *< 0.01). Click here for file

Additional file 2 **Differentially regulated genes between mature and T_1 _immature B cells**. Shown are the references describing B-cell associated gene functions or, if unknown, primary functions in other cell types, as listed in Table 1. Click here for file

Additional file 3 **Time-dependent changes in gene functions triggered by BCR stimulation in immature and mature B cells**. The most significant gene ontology categories for the genes discriminating between BCR-triggered (BCR) and control (ctrl) immature and mature B cells at 2 h are listed. Genes were identified by one-way ANOVA (*P *< 0.01). The *P*-value that is shown with each functional category indicates its statistical enrichment compared to the whole set of genes that were assayed with the microarrays. The significance of each category was evaluated using the modified Fisher's exact test employed by the DAVID functional annotation tool . The most significantly enriched functional categories in mature B cells have been highlighted in color to facilitate their viewing. Click here for file

Additional file 4 **BCR-regulated genes discriminating immature and mature B cells at 2 h**. Shown are the references describing B-cell associated gene functions or, if unknown, primary functions in other cell types, as listed in Table 2. Click here for file

Additional file 5 **Statistical analysis of *cis*-acting sequences in promoter regions of co-regulated genes**. (**A**) Co_clustering of the five focus gene, *Ptger4*, *Marcksl1*, *Myc*, *Crsp9 *and *Ifrd1*, by k-means analysis is independent of the selected k value. Each number in the table presents the identification number of a cluster. Note that the data from two different probes each (a, b) for *Marcksl1 *and *Myc *were used for clustering. (**B**) The significance of the predicted *Pax5 *and ETS family members' binding sites in the promoter regions of was evaluated using the Genomatix software (see also Figure 2B). Click here for file
