# Supplementary figures and images for: A Myc-regulated transcriptional network controls B-cell fate in response to BCR triggering
Source: BMC Genomics. 2009 Jul 17;10:323. doi: 10.1186/1471-2164-10-323 (PMC2722676; doi:10.1186/1471-2164-10-323)

A

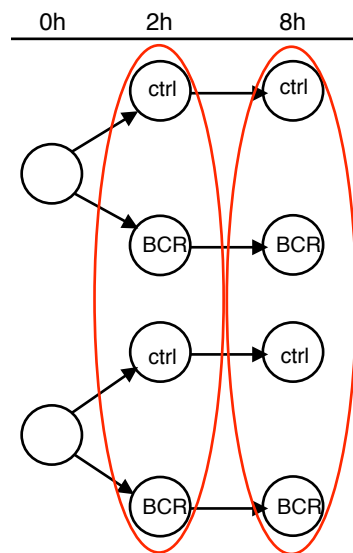

B

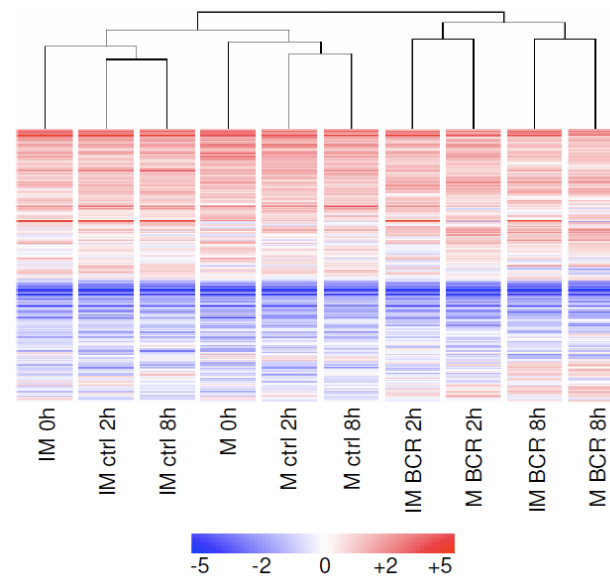

C

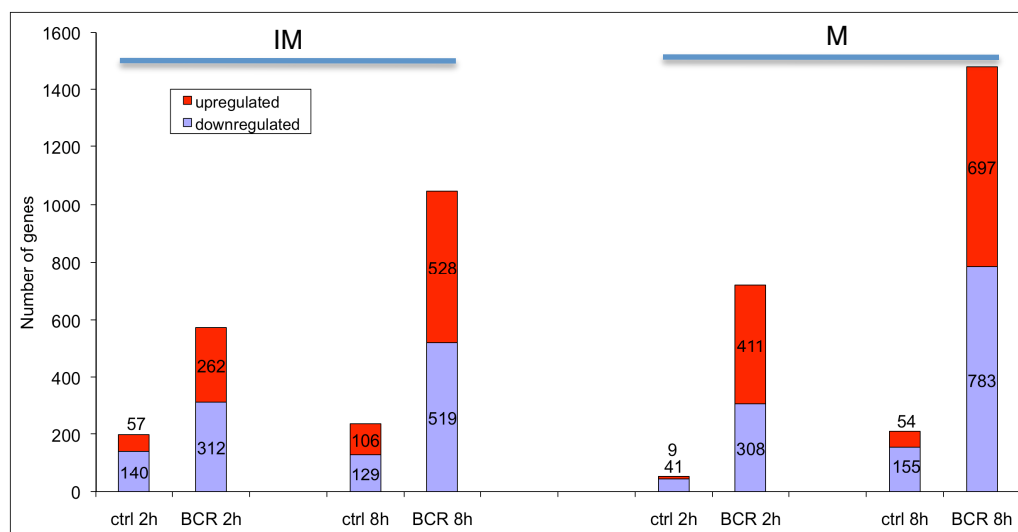

Supplement: Additional file 1 — Changes in gene expression underlying BCR-induced apoptosis and proliferation. (A) Experimental design used to identify the genes discriminating BCR-triggered transcriptional responses of immature and mature B cells at 2 and 8 h. Expression profiles used with two-way ANOVA are circled in red. Genes that passed the P < 0.05 criterion for each of the three parameters, which are maturity (M or IM), treatment (ctrl or BCR) and interaction between maturity and treatment, were identified as the discriminating genes for each time point (Table 1). (B) Unsupervised hierarchical cluster analysis of immature (IM) and mature (M) B cells of C57BL/6 mice both freshly isolated (0 h) and stimulated with (BCR) or without (ctrl) anti-F(ab')2 for 2 and 8 h. A hierarchical clustering algorithm based on standard correlations was applied in order to group all interrogated genes (13,043 gene features) on the basis of similarity in the pattern over all samples and all samples on the basis of similarity in the pattern over all genes. The data are presented in a matrix format, in which a column represents an individual sample and a row represents an individual gene. The red and blue colors in cells reflect high and low expression levels, respectively, as indicated in the scale bar (log-transformed scale). (C) Numbers of differentially expressed genes features in anti-IgM treated immature and mature B cells at 2 and 8 h versus the respective zero hour states (one-way ANOVA; P < 0.01). [file 1471-2164-10-323-S1.pdf]
