# Supplementary material for: A Myc-regulated transcriptional network controls B-cell fate in response to BCR triggering
Source: BMC Genomics. 2009 Jul 17;10:323. doi: 10.1186/1471-2164-10-323 (PMC2722676; doi:10.1186/1471-2164-10-323)
Supplement: Additional file 2 — Differentially regulated genes between mature and T1 immature B cells. Shown are the references describing B-cell associated gene functions or, if unknown, primary functions in other cell types, as listed in Table 1. [file 1471-2164-10-323-S2.pdf]

Gene symbol	Function/observations in B cells or other cell types	M/IM
Syngr2	B-cell and neuronal differentiation [1-3]	2.1
Idi1	cholesterol synthesis [4, 5]	1.7
Myc	B-cell proliferation, differentiation, apoptosis [6-9]	1.7
Litaf	regulates inflammatory cytokine expression [10, 11]	1.5
Spag9	positively regulates MAPK signaling pathways [12, 13]	1.4
Rnf5	inhibits cell motility [14]	1.4
Mfn2	mitochondrial fusion [15-17]	1.3
Txnip	immune cell differentiation [18]	1.3
Mfap3	no data	-1.3
Bnip3l	apoptosis [19-21]	-1.3
Clta	intracellular protein transport [22-24]	-1.5
Actb	cytoskeleton structure [25]	-1.5
Pik3c2a	survival and proliferation [26]	-1.6
Acad9	mitochondrial fatty acid oxidation [27]	-1.6
Prkcd	inhibits B-cell proliferation and differentiation [28, 29]	-1.6
Vezf1	angiogenesis [30, 31]	-1.6
Pcyt1a	apoptosis [32]	-1.7
Cecr2	chromatin remodeling [33]	-1.7
Zcchc17	no data	-1.7
Coro1b	neurite outgrowth [34]	-1.7
Atp5c1	no data	-1.8
H2afz	embryonic development [35]	-1.8
Cd24a	B-cell apoptosis; development of immature B cells [36-38]	-2.1
Npc2	cholesterol transport [39]	-2.1
Emb	cell adhesion during development [40]	-2.2
Fcgr2b	inhibits B-cell proliferation [41]	-2.2
Emx2	neuronal growth and development [42, 43]	-2.3
Cst3	reduces antigen presenting capacity of dendritic cells [44]	-2.3
Id2	inhibits B-cell differentiation [45, 46]	-2.6
Cul7	regulates B-cell differentiation [47]	-2.6
Wwp1	T-cell differentiation; embryogenesis [48, 49]	-2.7
Jun	apoptosis, proliferation, differentiation [50-52]	-2.7
Ctsb	apoptosis [53-55]	-2.8
Ifitm3	inhibits proliferation [56, 57]	-3.1
Nr2f2	regulates organogenesis [58-60]	-3.1
Atp1b1	inhibits T-cell activation [61]	-3.2
Slc40a1	iron export [62]	-3.6
Phtf	regulates spermatogenesis [63, 64]	-3.7
Hoxb3	regeneration of stem cells [65-67]	-3.8
C1qa	reduces T cell-dependent immune response [68-70]	-5.1
Laptm5	B-cell differentiation; inhibits maturation of dendritic cells [71, 72]	-6.2
Hmox1	inhibits B-cell activation; inhibits T-cell proliferation [73-75]	-7.4
Hba-a1	oxygen transport [76]	-7.9
Lyzs	defense response to bacteria [77-79]	-23.8

References

1. Schroder AJ, Pavlidis P, Arimura A, Capece D, Rothman PB: **Cutting edge: STAT6 serves as a positive and negative regulator of gene expression in IL-4-stimulated B lymphocytes**. *J Immunol* 2002, **168**(3):996-1000.
2. Wang CC, Kadota M, Nishigaki R, Kazuki Y, Shirayoshi Y, Rogers MS, Gojobori T, Ikeo K, Oshimura M: **Molecular hierarchy in neurons differentiated from mouse ES cells containing a single human chromosome 21**. *Biochem Biophys Res Commun* 2004, **314**(2):335-350.
3. Belfort GM, Kandror KV: **Cellugyrin and synaptogyrin facilitate targeting of synaptophysin to a ubiquitous synaptic vesicle-sized compartment in PC12 cells**. *J Biol Chem* 2003, **278**(48):47971-47978.
4. Stolarov J, Chang K, Reiner A, Rodgers L, Hannon GJ, Wigler MH, Mittal V: **Design of a retroviral-mediated ecdysone-inducible system and its application to the expression profiling of the PTEN tumor suppressor**. *Proc Natl Acad Sci U S A* 2001, **98**(23):13043-13048.
5. Sweeney C, Fambrough D, Huard C, Diamonti AJ, Lander ES, Cantley LC, Carraway KL, 3rd: **Growth factor-specific signaling pathway stimulation and gene expression mediated by ErbB receptors**. *J Biol Chem* 2001, **276**(25):22685-22698.
6. de Alboran IM, O'Hagan RC, Gartner F, Malynn B, Davidson L, Rickert R, Rajewsky K, DePinho RA, Alt FW: **Analysis of C-MYC function in normal cells via conditional gene-targeted mutation**. *Immunity* 2001, **14**(1):45-55.
7. Iritani BM, Eisenman RN: **c-Myc enhances protein synthesis and cell size during B lymphocyte development**. *Proc Natl Acad Sci U S A* 1999, **96**(23):13180-13185.
8. Lin Y, Wong K, Calame K: **Repression of c-myc transcription by Blimp-1, an inducer of terminal B cell differentiation**. *Science* 1997, **276**(5312):596-599.
9. Lin KI, Lin Y, Calame K: **Repression of c-myc is necessary but not sufficient for terminal differentiation of B lymphocytes in vitro**. *Mol Cell Biol* 2000, **20**(23):8684-8695.
10. Myokai F, Takashiba S, Lebo R, Amar S: **A novel lipopolysaccharide-induced transcription factor regulating tumor necrosis factor alpha gene expression: molecular cloning, sequencing, characterization, and chromosomal assignment**. *Proc Natl Acad Sci U S A* 1999, **96**(8):4518-4523.
11. Tang X, Marciano DL, Leeman SE, Amar S: **LPS induces the interaction of a transcription factor, LPS-induced TNF-alpha factor, and STAT6(B) with effects on multiple cytokines**. *Proc Natl Acad Sci U S A* 2005, **102**(14):5132-5137.
12. Lee CM, Onesime D, Reddy CD, Dhanasekaran N, Reddy EP: **JLP: A scaffolding protein that tethers JNK/p38MAPK signaling modules and transcription factors**. *Proc Natl Acad Sci U S A* 2002, **99**(22):14189-14194.
13. Kelkar N, Standen CL, Davis RJ: **Role of the JIP4 scaffold protein in the regulation of mitogen-activated protein kinase signaling pathways**. *Mol Cell Biol* 2005, **25**(7):2733-2743.
14. Didier C, Broday L, Bhoumik A, Israeli S, Takahashi S, Nakayama K, Thomas SM, Turner CE, Henderson S, Sabe H *et al*: **RNF5, a RING finger protein that regulates cell motility by targeting paxillin ubiquitination and altered localization**. *Mol Cell Biol* 2003, **23**(15):5331-5345.
15. Honda S, Aihara T, Hontani M, Okubo K, Hirose S: **Mutational analysis of action of mitochondrial fusion factor mitofusin-2**. *J Cell Sci* 2005, **118**(Pt 14):3153-3161.
16. Koshiba T, Detmer SA, Kaiser JT, Chen H, McCaffery JM, Chan DC: **Structural basis of mitochondrial tethering by mitofusin complexes**. *Science* 2004, **305**(5685):858-862.
17. Chen H, Detmer SA, Ewald AJ, Griffin EE, Fraser SE, Chan DC: **Mitofusins Mfn1 and Mfn2 coordinately regulate mitochondrial fusion and are essential for embryonic development**. *J Cell Biol* 2003, **160**(2):189-200.
18. Lee KN, Kang HS, Jeon JH, Kim EM, Yoon SR, Song H, Lyu CY, Piao ZH, Kim SU, Han YH *et al*: **VDUP1 is required for the development of natural killer cells**. *Immunity* 2005, **22**(2):195-208.
19. Chen G, Ray R, Dubik D, Shi L, Cizeau J, Bleackley RC, Saxena S, Gietz RD, Greenberg AH: **The E1B 19K/Bcl-2-binding protein Nip3 is a dimeric mitochondrial protein that activates apoptosis**. *J Exp Med* 1997, **186**(12):1975-1983.
20. Vande Velde C, Cizeau J, Dubik D, Alimonti J, Brown T, Israels S, Hakem R, Greenberg AH: **BNIP3 and genetic control of necrosis-like cell death through the mitochondrial permeability transition pore**. *Mol Cell Biol* 2000, **20**(15):5454-5468.
21. Aoyagi M, Higashino F, Yasuda M, Takahashi A, Sawada Y, Totsuka Y, Kohgo T, Sano H, Kobayashi M, Shindoh M: **Nuclear export of adenovirus E4orf6 protein is necessary for its ability to antagonize apoptotic activity of BH3-only proteins**. *Oncogene* 2003, **22**(44):6919-6927.
22. Walther K, Krauss M, Diril MK, Lemke S, Ricotta D, Honing S, Kaiser S, Haucke V: **Human stoned B interacts with AP-2 and synaptotagmin and facilitates clathrin-coated vesicle uncoating**. *EMBO Rep* 2001, **2**(7):634-640.

23. Blondeau F, Ritter B, Allaire PD, Wasiak S, Girard M, Hussain NK, Angers A, Legendre-Guillemin V, Roy L, Boismenu D *et al*: **Tandem MS analysis of brain clathrin-coated vesicles reveals their critical involvement in synaptic vesicle recycling**. *Proc Natl Acad Sci U S A* 2004, **101**(11):3833-3838.
24. Wasiak S, Legendre-Guillemin V, Puertollano R, Blondeau F, Girard M, de Heuvel E, Boismenu D, Bell AW, Bonifacino JS, McPherson PS: **Enthoprotin: a novel clathrin-associated protein identified through subcellular proteomics**. *J Cell Biol* 2002, **158**(5):855-862.
25. Schevzov G, Lloyd C, Gunning P: **High level expression of transfected beta- and gamma-actin genes differentially impacts on myoblast cytoarchitecture**. *J Cell Biol* 1992, **117**(4):775-785.
26. Misawa H, Ohtsubo M, Copeland NG, Gilbert DJ, Jenkins NA, Yoshimura A: **Cloning and characterization of a novel class II phosphoinositide 3-kinase containing C2 domain**. *Biochem Biophys Res Commun* 1998, **244**(2):531-539.
27. He M, Rutledge SL, Kelly DR, Palmer CA, Murdoch G, Majumder N, Nicholls RD, Pei Z, Watkins PA, Vockley J: **A new genetic disorder in mitochondrial fatty acid beta-oxidation: ACAD9 deficiency**. *Am J Hum Genet* 2007, **81**(1):87-103.
28. Mecklenbrauker I, Saijo K, Zheng NY, Leitges M, Tarakhovsky A: **Protein kinase Cdelta controls self-antigen-induced B-cell tolerance**. *Nature* 2002, **416**(6883):860-865.
29. Miyamoto A, Nakayama K, Imaki H, Hirose S, Jiang Y, Abe M, Tsukiyama T, Nagahama H, Ohno S, Hatakeyama S *et al*: **Increased proliferation of B cells and auto-immunity in mice lacking protein kinase Cdelta**. *Nature* 2002, **416**(6883):865-869.
30. Park IK, He Y, Lin F, Laerum OD, Tian Q, Bumgarner R, Klug CA, Li K, Kuhr C, Doyle MJ *et al*: **Differential gene expression profiling of adult murine hematopoietic stem cells**. *Blood* 2002, **99**(2):488-498.
31. Aitsebaomo J, Kingsley-Kallesen ML, Wu Y, Quertermous T, Patterson C: **Vezf1/DB1 is an endothelial cell-specific transcription factor that regulates expression of the endothelin-1 promoter**. *J Biol Chem* 2001, **276**(42):39197-39205.
32. Zhang D, Tang W, Yao PM, Yang C, Xie B, Jackowski S, Tabas I: **Macrophages deficient in CTP:Phosphocholine cytidylyltransferase-alpha are viable under normal culture conditions but are highly susceptible to free cholesterol-induced death. Molecular genetic evidence that the induction of phosphatidylcholine biosynthesis in free cholesterol-loaded macrophages is an adaptive response**. *J Biol Chem* 2000, **275**(45):35368-35376.
33. Banting GS, Barak O, Ames TM, Burnham AC, Kardel MD, Cooch NS, Davidson CE, Godbout R, McDermid HE, Shiekhattar R: **CECR2, a protein involved in neurulation, forms a novel chromatin remodeling complex with SNF2L**. *Hum Mol Genet* 2005, **14**(4):513-524.
34. Di Giovanni S, De Biase A, Yakovlev A, Finn T, Beers J, Hoffman EP, Faden AI: **In vivo and in vitro characterization of novel neuronal plasticity factors identified following spinal cord injury**. *J Biol Chem* 2005, **280**(3):2084-2091.
35. Faast R, Thonglairoam V, Schulz TC, Beall J, Wells JR, Taylor H, Matthaei K, Rathjen PD, Tremethick DJ, Lyons I: **Histone variant H2A.Z is required for early mammalian development**. *Curr Biol* 2001, **11**(15):1183-1187.
36. Nielsen PJ, Lorenz B, Muller AM, Wenger RH, Brombacher F, Simon M, von der Weid T, Langhorne WJ, Mossmann H, Kohler G: **Altered erythrocytes and a leaky block in B-cell development in CD24/HSA-deficient mice**. *Blood* 1997, **89**(3):1058-1067.
37. Suzuki T, Kiyokawa N, Taguchi T, Sekino T, Katagiri YU, Fujimoto J: **CD24 induces apoptosis in human B cells via the glycolipid-enriched membrane domains/rafts-mediated signaling system**. *J Immunol* 2001, **166**(9):5567-5577.
38. Wenger RH, Rochelle JM, Seldin MF, Kohler G, Nielsen PJ: **The heat stable antigen (mouse CD24) gene is differentially regulated but has a housekeeping promoter**. *J Biol Chem* 1993, **268**(31):23345-23352.
39. Naureckiene S, Sleat DE, Lackland H, Fensom A, Vanier MT, Wattiaux R, Jadot M, Lobel P: **Identification of HE1 as the second gene of Niemann-Pick C disease**. *Science* 2000, **290**(5500):2298-2301.
40. Guenette RS, Sridhar S, Herley M, Mooibroek M, Wong P, Tenniswood M: **Embigin, a developmentally expressed member of the immunoglobulin super family, is also expressed during regression of prostate and mammary gland**. *Dev Genet* 1997, **21**(4):268-278.
41. Takai T, Ono M, Hikida M, Ohmori H, Ravetch JV: **Augmented humoral and anaphylactic responses in Fc gamma RII-deficient mice**. *Nature* 1996, **379**(6563):346-349.
42. Mallamaci A, Muzio L, Chan CH, Parnavelas J, Boncinelli E: **Area identity shifts in the early cerebral cortex of Emx2-/- mutant mice**. *Nat Neurosci* 2000, **3**(7):679-686.
43. Galli R, Fiocco R, De Filippis L, Muzio L, Gritti A, Mercurio S, Broccoli V, Pellegrini M, Mallamaci A, Vescovi AL: **Emx2 regulates the proliferation of stem cells of the adult mammalian central nervous system**. *Development* 2002, **129**(7):1633-1644.

44. Pierre P, Mellman I: **Developmental regulation of invariant chain proteolysis controls MHC class II trafficking in mouse dendritic cells**. *Cell* 1998, **93**(7):1135-1145.
45. Becker-Herman S, Lantner F, Shachar I: **Id2 negatively regulates B cell differentiation in the spleen**. *J Immunol* 2002, **168**(11):5507-5513.
46. Nilsson JA, Nilsson LM, Keller U, Yokota Y, Boyd K, Cleveland JL: **Id2 is dispensable for myc-induced lymphomagenesis**. *Cancer Res* 2004, **64**(20):7296-7301.
47. Zhan F, Tian E, Bumm K, Smith R, Barlogie B, Shaughnessy J, Jr.: **Gene expression profiling of human plasma cell differentiation and classification of multiple myeloma based on similarities to distinct stages of late-stage B-cell development**. *Blood* 2003, **101**(3):1128-1140.
48. Huang K, Johnson KD, Petcherski AG, Vandergon T, Mosser EA, Copeland NG, Jenkins NA, Kimble J, Bresnick EH: **A HECT domain ubiquitin ligase closely related to the mammalian protein WWP1 is essential for Caenorhabditis elegans embryogenesis**. *Gene* 2000, **252**(1-2):137-145.
49. Deng YJ, Huang ZX, Zhou CJ, Wang JW, You Y, Song ZQ, Xiang MM, Zhong BY, Hao F: **Gene profiling involved in immature CD4+ T lymphocyte responsible for systemic lupus erythematosus**. *Mol Immunol* 2006, **43**(9):1497-1507.
50. Bossy-Wetzel E, Bakiri L, Yaniv M: **Induction of apoptosis by the transcription factor c-Jun**. *Embo J* 1997, **16**(7):1695-1709.
51. Schreiber M, Kolbus A, Piu F, Szabowski A, Mohle-Steinlein U, Tian J, Karin M, Angel P, Wagner EF: **Control of cell cycle progression by c-Jun is p53 dependent**. *Genes Dev* 1999, **13**(5):607-619.
52. Chen J, Stewart V, Spyrou G, Hilberg F, Wagner EF, Alt FW: **Generation of normal T and B lymphocytes by c-jun deficient embryonic stem cells**. *Immunity* 1994, **1**(1):65-72.
53. Guicciardi ME, Deussing J, Miyoshi H, Bronk SF, Svingen PA, Peters C, Kaufmann SH, Gores GJ: **Cathepsin B contributes to TNF-alpha-mediated hepatocyte apoptosis by promoting mitochondrial release of cytochrome c**. *J Clin Invest* 2000, **106**(9):1127-1137.
54. Foghsgaard L, Wissing D, Mauch D, Lademann U, Bastholm L, Boes M, Elling F, Leist M, Jaattela M: **Cathepsin B acts as a dominant execution protease in tumor cell apoptosis induced by tumor necrosis factor**. *J Cell Biol* 2001, **153**(5):999-1010.
55. Broker LE, Huisman C, Span SW, Rodriguez JA, Kruyt FA, Giaccone G: **Cathepsin B mediates caspase-independent cell death induced by microtubule stabilizing agents in non-small cell lung cancer cells**. *Cancer Res* 2004, **64**(1):27-30.
56. Ropolo A, Tomasini R, Grasso D, Dusetti NJ, Cerquetti MC, Iovanna JL, Vaccaro MI: **Cloning of IP15, a pancreatitis-induced gene whose expression inhibits cell growth**. *Biochem Biophys Res Commun* 2004, **319**(3):1001-1009.
57. Brem R, Oraszlan-Szovik K, Foser S, Bohrmann B, Certa U: **Inhibition of proliferation by 1-8U in interferon-alpha-responsive and non-responsive cell lines**. *Cell Mol Life Sci* 2003, **60**(6):1235-1248.
58. Pereira FA, Qiu Y, Zhou G, Tsai MJ, Tsai SY: **The orphan nuclear receptor COUP-TFII is required for angiogenesis and heart development**. *Genes Dev* 1999, **13**(8):1037-1049.
59. Tripodi M, Filosa A, Armentano M, Studer M: **The COUP-TF nuclear receptors regulate cell migration in the mammalian basal forebrain**. *Development* 2004, **131**(24):6119-6129.
60. Lee CT, Li L, Takamoto N, Martin JF, Demayo FJ, Tsai MJ, Tsai SY: **The nuclear orphan receptor COUP-TFII is required for limb and skeletal muscle development**. *Mol Cell Biol* 2004, **24**(24):10835-10843.
61. Teague TK, Hildeman D, Kedl RM, Mitchell T, Rees W, Schaefer BC, Bender J, Kappler J, Marrack P: **Activation changes the spectrum but not the diversity of genes expressed by T cells**. *Proc Natl Acad Sci U S A* 1999, **96**(22):12691-12696.
62. Donovan A, Brownlie A, Zhou Y, Shepard J, Pratt SJ, Moynihan J, Paw BH, Drejer A, Barut B, Zapata A *et al*: **Positional cloning of zebrafish ferroportin1 identifies a conserved vertebrate iron exporter**. *Nature* 2000, **403**(6771):776-781.
63. Oyhenart J, Le Goffic R, Samson M, Jegou B, Raich N: **Phtf1 is an integral membrane protein localized in an endoplasmic reticulum domain in maturing male germ cells**. *Biol Reprod* 2003, **68**(3):1044-1053.
64. Manuel A, Beaupain D, Romeo PH, Raich N: **Molecular characterization of a novel gene family (PHTF) conserved from Drosophila to mammals**. *Genomics* 2000, **64**(2):216-220.
65. Bjornsson JM, Larsson N, Brun AC, Magnusson M, Andersson E, Lundstrom P, Larsson J, Repetowska E, Ehinger M, Humphries RK *et al*: **Reduced proliferative capacity of hematopoietic stem cells deficient in Hoxb3 and Hoxb4**. *Mol Cell Biol* 2003, **23**(11):3872-3883.
66. Mizusawa N, Hasegawa T, Ohigashi I, Tanaka-Kosugi C, Harada N, Itakura M, Yoshimoto K: **Differentiation phenotypes of pancreatic islet beta- and alpha-cells are closely related with homeotic genes and a group of differentially expressed genes**. *Gene* 2004, **331**:53-63.

67. Yoshimi T, Nakamura N, Shimada S, Iguchi K, Hashimoto F, Mochitate K, Takahashi Y, Miura T: **Homeobox B3, FoxA1 and FoxA2 interactions in epithelial lung cell differentiation of the multipotent M3E3/C3 cell line**. *Eur J Cell Biol* 2005, **84**(5):555-566.
68. Cutler AJ, Botto M, van Essen D, Rivi R, Davies KA, Gray D, Walport MJ: **T cell-dependent immune response in C1q-deficient mice: defective interferon gamma production by antigen-specific T cells**. *J Exp Med* 1998, **187**(11):1789-1797.
69. Trendelenburg M, Manderson AP, Fossati-Jimack L, Walport MJ, Botto M: **Monocytosis and accelerated activation of lymphocytes in C1q-deficient autoimmune-prone mice**. *Immunology* 2004, **113**(1):80-88.
70. Stager S, Alexander J, Kirby AC, Botto M, Rooijen NV, Smith DF, Brombacher F, Kaye PM: **Natural antibodies and complement are endogenous adjuvants for vaccine-induced CD8+ T-cell responses**. *Nat Med* 2003, **9**(10):1287-1292.
71. Seimiya M, J OW, Bahar R, Kawamura K, Wang Y, Saisho H, Tagawa M: **Stage-specific expression of Clast6/E3/LAPTM5 during B cell differentiation: elevated expression in human B lymphomas**. *Int J Oncol* 2003, **22**(2):301-304.
72. Hashimoto SI, Suzuki T, Nagai S, Yamashita T, Toyoda N, Matsushima K: **Identification of genes specifically expressed in human activated and mature dendritic cells through serial analysis of gene expression**. *Blood* 2000, **96**(6):2206-2214.
73. Kapturczak MH, Wasserfall C, Brusko T, Campbell-Thompson M, Ellis TM, Atkinson MA, Agarwal A: **Heme oxygenase-1 modulates early inflammatory responses: evidence from the heme oxygenase-1-deficient mouse**. *Am J Pathol* 2004, **165**(3):1045-1053.
74. Pae HO, Oh GS, Choi BM, Chae SC, Kim YM, Chung KR, Chung HT: **Carbon monoxide produced by heme oxygenase-1 suppresses T cell proliferation via inhibition of IL-2 production**. *J Immunol* 2004, **172**(8):4744-4751.
75. Hori R, Kashiba M, Toma T, Yachie A, Goda N, Makino N, Soejima A, Nagasawa T, Nakabayashi K, Suematsu M: **Gene transfection of H25A mutant heme oxygenase-1 protects cells against hydroperoxide-induced cytotoxicity**. *J Biol Chem* 2002, **277**(12):10712-10718.
76. Paszty C, Mohandas N, Stevens ME, Loring JF, Liebhaber SA, Brion CM, Rubin EM: **Lethal alpha-thalassaemia created by gene targeting in mice and its genetic rescue**. *Nat Genet* 1995, **11**(1):33-39.
77. Cross M, Mangelsdorf I, Wedel A, Renkawitz R: **Mouse lysozyme M gene: isolation, characterization, and expression studies**. *Proc Natl Acad Sci U S A* 1988, **85**(17):6232-6236.
78. Faust N, Varas F, Kelly LM, Heck S, Graf T: **Insertion of enhanced green fluorescent protein into the lysozyme gene creates mice with green fluorescent granulocytes and macrophages**. *Blood* 2000, **96**(2):719-726.
79. Brady G, Billia F, Knox J, Hoang T, Kirsch IR, Voura EB, Hawley RG, Cumming R, Buchwald M, Siminovitch K: **Analysis of gene expression in a complex differentiation hierarchy by global amplification of cDNA from single cells**. *Curr Biol* 1995, **5**(8):909-922.
