# Supplementary material for: A Myc-regulated transcriptional network controls B-cell fate in response to BCR triggering
Source: BMC Genomics. 2009 Jul 17;10:323. doi: 10.1186/1471-2164-10-323 (PMC2722676; doi:10.1186/1471-2164-10-323)
Supplement: Additional file 3 — Time-dependent changes in gene functions triggered by BCR stimulation in immature and mature B cells. The most significant gene ontology categories for the genes discriminating between BCR-triggered (BCR) and control (ctrl) immature and mature B cells at 2 h are listed. Genes were identified by one-way ANOVA (P < 0.01). The P-value that is shown with each functional category indicates its statistical enrichment compared to the whole set of genes that were assayed with the microarrays. The significance of each category was evaluated using the modified Fisher's exact test employed by the DAVID functional annotation tool . The most significantly enriched functional categories in mature B cells have been highlighted in color to facilitate their viewing. [file 1471-2164-10-323-S3.pdf]

Immature at 2h (BCR vs. ctrl)

GO category	P-value
response to temperature stimulus	6.08E-04
response to heat	0.0015
protein folding	0.0058
positive regulation of nitric oxide biosynthetic process	0.0095
G-protein signaling, coupled to cAMP nucleotide second messenger	0.0176
intracellular signaling cascade	0.0197
G-protein signaling, coupled to cyclic nucleotide second messenger	0.0214
response to stimulus	0.0236
regulation of a molecular function	0.0240
cellular process	0.0275
response to stress	0.0293
cAMP-mediated signaling	0.0301
regulation of nitric oxide biosynthetic process	0.0306
response to abiotic stimulus	0.0308
biosynthetic process	0.0331
ribonucleoprotein complex biogenesis and assembly	0.0346
cyclic-nucleotide-mediated signaling	0.0351
biological regulation	0.0399
regulation of catalytic activity	0.0417
small GTPase mediated signal transduction	0.0451
negative regulation of locomotion	0.0497
negative regulation of cell motility	0.0497
cellular metabolic process	0.0499
ribosome biogenesis and assembly	0.0553
cofactor metabolic process	0.0592
one-carbon compound metabolic process	0.0601
G-protein signaling, adenylate cyclase activating pathway	0.0605
coenzyme metabolic process	0.0613
cell aging	0.0720
second-messenger-mediated signaling	0.0793
cellular lipid metabolic process	0.0797

Mature at 2h (BCR vs. ctrl)

GO category	P-value
ribonucleoprotein complex biogenesis and assembly	2.11E-08
ribosome biogenesis and assembly	5.76E-08
biosynthetic process	7.30E-07
cellular biosynthetic process	9.50E-06
rRNA processing	1.47E-05
rRNA metabolic process	2.01E-05
gene expression	2.89E-05
metabolic process	4.97E-05
cellular metabolic process	1.06E-04
response to temperature stimulus	1.51E-04
translation	2.75E-04
cellular process	3.28E-04
sterol biosynthetic process	5.77E-04
macromolecule biosynthetic process	7.86E-04
cholesterol biosynthetic process	9.78E-04
primary metabolic process	0.0011
sterol metabolic process	0.0012
response to heat	0.0015
cellular component organization and biogenesis	0.0018
cholesterol metabolic process	0.0024
regulation of biosynthetic process	0.0024
protein folding	0.0025
regulation of catalytic activity	0.0042
regulation of a molecular function	0.0048
oligosaccharide metabolic process	0.0063
hemopoietic or lymphoid organ development	0.0068
regulation of nitric oxide biosynthetic process	0.0104
organelle organization and biogenesis	0.0107
immune system development	0.0127
positive regulation of nucleotide and nucleic acid metal	0.0131
rRNA modification	0.0148
