# Supplementary material for: A Myc-regulated transcriptional network controls B-cell fate in response to BCR triggering
Source: BMC Genomics. 2009 Jul 17;10:323. doi: 10.1186/1471-2164-10-323 (PMC2722676; doi:10.1186/1471-2164-10-323)
Supplement: Additional file 4 — BCR-regulated genes discriminating immature and mature B cells at 2 h. Shown are the references describing B-cell associated gene functions or, if unknown, primary functions in other cell types, as listed in Table 2. [file 1471-2164-10-323-S4.pdf]

		microarrays			qPCR	
		BCR/ctrl			BCR/ctrl	
Gene symbol	Function	IM	M	M/IM	IM	M
Ptger4	B-cell activation and differentiation [1, 2]	1.7	4.6	**2.8**	1.8	4.8
Marcksl1	proliferation, cell adhesion, neurosecretion [3-8]	2.6	6.1	**2.4**	2.2	4.3
Myc	B-cell proliferation, differentiation, apoptosis [9-12]	3.8	8.9	**2.3**	4.1	12.9
Crsp9	Proliferation [13-16]	1.4	3.0	**2.1**	1.7	3.1
Chchd4	no data	1.6	3.3	**2.1**	1.7	3.1
Wdr55	rRNA synthesis, cell cycle progression [17]	1.3	2.4	**1.9**	1.7	1.9
Ifrd1	differentiation, proliferation [18-22]	1.3	2.2	**1.7**	1.4	2.3
Eif3s1	T-cell activation [23]	1.3	2.3	**1.7**	1.7	2.0
Idi1	cholesterol synthesis [24, 25]	1.6	2.7	**1.7**	1.6	2.3
M6pr	lysosomal enzyme transport [26-28]	1.4	2.5	**1.7**	1.1	2.0
Nola2	T-cell activation [29]	1.4	2.3	**1.7**	1.7	2.2
Pdhx	electron transport [30, 31]	1.8	3.0	**1.7**	-1.3	-1.4
Hrb	spermiogenesis [32]	1.5	2.3	**1.5**	1.1	2.5
Lyar	cell growth [33]	1.3	1.9	**1.5**	1.2	2.7
Irx3	heart and CNS development [34-37]	-1.0	1.4	**1.4**	-1.2	1.5
Rpo1-3	cell growth [38, 39]	1.1	1.5	**1.4**	1.1	1.5
Ptdss1	cell growth [40, 41]	-1.0	1.4	**1.4**	-1.4	1.4
Stt3b	oligosaccharyltransferase activity [42]	1.9	1.3	**-1.5**	2.6	-1.3
Plekha2	B-cell receptor signaling [42-44]	-1.7	-2.6	**-1.6**	-2.5	-5.4
Fbxo22	no data	1.0	-1.6	**-1.6**	-1.4	-2.0
Jun	apoptosis, proliferation, differentiation [45-52]	1.1	-1.6	**-1.8**	-1.1	-3.4
3110001A13Rik	no data	-1.7	-3.6	**-2.1**	-1.6	-5.1
Spata13	no data	-1.8	-4.8	**-2.7**	-2.5	-16.7
Tmem23	apoptosis [53]	-1.9	-5.3	**-2.8**	-2.8	-20.0

References:

1. Fedyk ER, Phipps RP: **Prostaglandin E2 receptors of the EP2 and EP4 subtypes regulate activation and differentiation of mouse B lymphocytes to IgE-secreting cells**. *Proc Natl Acad Sci U S A* 1996, **93**(20):10978-10983.
2. Fedyk ER, Ripper JM, Brown DM, Phipps RP: **A molecular analysis of PGE receptor (EP) expression on normal and transformed B lymphocytes: coexpression of EP1, EP2, EP3beta and EP4**. *Mol Immunol* 1996, **33**(1):33-45.
3. Bonner AE, Lemon WJ, Devereux TR, Lubet RA, You M: **Molecular profiling of mouse lung tumors: association with tumor progression, lung development, and human lung adenocarcinomas**. *Oncogene* 2004, **23**(5):1166-1176.

4. Chang S, Hemmings HC, Jr., Aderem A: **Stimulus-dependent phosphorylation of MacMARCKS, a protein kinase C substrate, in nerve termini and PC12 cells**. *J Biol Chem* 1996, **271**(2):1174-1178.
5. Chen J, Chang S, Duncan SA, Okano HJ, Fishell G, Aderem A: **Disruption of the MacMARCKS gene prevents cranial neural tube closure and results in anencephaly**. *Proc Natl Acad Sci U S A* 1996, **93**(13):6275-6279.
6. Ono K, Tanaka T, Tsunoda T, Kitahara O, Kihara C, Okamoto A, Ochiai K, Takagi T, Nakamura Y: **Identification by cDNA microarray of genes involved in ovarian carcinogenesis**. *Cancer Res* 2000, **60**(18):5007-5011.
7. Yue L, Lu S, Garces J, Jin T, Li J: **Protein kinase C-regulated dynamitin-macrophage-enriched myristoylated alanine-rice C kinase substrate interaction is involved in macrophage cell spreading**. *J Biol Chem* 2000, **275**(31):23948-23956.
8. Zhou X, Li J: **Macrophage-enriched myristoylated alanine-rich C kinase substrate and its phosphorylation is required for the phorbol ester-stimulated diffusion of beta 2 integrin molecules**. *J Biol Chem* 2000, **275**(26):20217-20222.
9. de Alboran IM, O'Hagan RC, Gartner F, Malynn B, Davidson L, Rickert R, Rajewsky K, DePinho RA, Alt FW: **Analysis of C-MYC function in normal cells via conditional gene-targeted mutation**. *Immunity* 2001, **14**(1):45-55.
10. Iritani BM, Eisenman RN: **c-Myc enhances protein synthesis and cell size during B lymphocyte development**. *Proc Natl Acad Sci U S A* 1999, **96**(23):13180-13185.
11. Lin KI, Lin Y, Calame K: **Repression of c-myc is necessary but not sufficient for terminal differentiation of B lymphocytes in vitro**. *Mol Cell Biol* 2000, **20**(23):8684-8695.
12. Lin Y, Wong K, Calame K: **Repression of c-myc transcription by Blimp-1, an inducer of terminal B cell differentiation**. *Science* 1997, **276**(5312):596-599.
13. Baldacchino V, Oble S, Decarie PO, Bourdeau I, Hamet P, Tremblay J, Lacroix A: **The Sp transcription factors are involved in the cellular expression of the human glucose-dependent insulinotropic polypeptide receptor gene and overexpressed in adrenals of patients with Cushing's syndrome**. *J Mol Endocrinol* 2005, **35**(1):61-71.
14. Kwon JY, Park JM, Gim BS, Han SJ, Lee J, Kim YJ: **Caenorhabditis elegans mediator complexes are required for developmental-specific transcriptional activation**. *Proc Natl Acad Sci U S A* 1999, **96**(26):14990-14995.
15. Myers LC, Gustafsson CM, Bushnell DA, Lui M, Erdjument-Bromage H, Tempst P, Kornberg RD: **The Med proteins of yeast and their function through the RNA polymerase II carboxy-terminal domain**. *Genes Dev* 1998, **12**(1):45-54.
16. Ryu S, Zhou S, Ladurner AG, Tjian R: **The transcriptional cofactor complex CRSP is required for activity of the enhancer-binding protein Sp1**. *Nature* 1999, **397**(6718):446-450.
17. Iwanami N, Higuchi T, Sasano Y, Fujiwara T, Hoa VQ, Okada M, Talukder SR, Kunimatsu S, Li J, Saito F *et al*: **WDR55 is a nucleolar modulator of ribosomal RNA synthesis, cell cycle progression, and teleost organ development**. *PLoS Genet* 2008, **4**(8):e1000171.
18. Micheli L, Leonardi L, Conti F, Buanne P, Canu N, Caruso M, Tirone F: **PC4 coactivates MyoD by relieving the histone deacetylase 4-mediated inhibition of myocyte enhancer factor 2C**. *Mol Cell Biol* 2005, **25**(6):2242-2259.
19. Rubin DC, Swietlicki EA, Wang JL, Levin MS: **Regulation of PC4/TIS7 expression in adapting remnant intestine after resection**. *Am J Physiol* 1998, **275**(3 Pt 1):G506-513.

20. Vadivelu SK, Kurzbauer R, Dieplinger B, Zweyer M, Schafer R, Wernig A, Vietor I, Huber LA: **Muscle regeneration and myogenic differentiation defects in mice lacking TIS7**. *Mol Cell Biol* 2004, **24**(8):3514-3525.
21. Varnum BC, Lim RW, Kujubu DA, Luner SJ, Kaufman SE, Greenberger JS, Gasson JC, Herschman HR: **Granulocyte-macrophage colony-stimulating factor and tetradecanoyl phorbol acetate induce a distinct, restricted subset of primary-response TIS genes in both proliferating and terminally differentiated myeloid cells**. *Mol Cell Biol* 1989, **9**(8):3580-3583.
22. Vietor I, Vadivelu SK, Wick N, Hoffman R, Cotten M, Seiser C, Fialka I, Wunderlich W, Haase A, Korinkova G *et al*: **TIS7 interacts with the mammalian SIN3 histone deacetylase complex in epithelial cells**. *Embo J* 2002, **21**(17):4621-4631.
23. Mizusawa N, Hasegawa T, Ohigashi I, Tanaka-Kosugi C, Harada N, Itakura M, Yoshimoto K: **Differentiation phenotypes of pancreatic islet beta- and alpha-cells are closely related with homeotic genes and a group of differentially expressed genes**. *Gene* 2004, **331**:53-63.
24. Stolarov J, Chang K, Reiner A, Rodgers L, Hannon GJ, Wigler MH, Mittal V: **Design of a retroviral-mediated ecdysone-inducible system and its application to the expression profiling of the PTEN tumor suppressor**. *Proc Natl Acad Sci U S A* 2001, **98**(23):13043-13048.
25. Sweeney C, Fambrough D, Huard C, Diamonti AJ, Lander ES, Cantley LC, Carraway KL, 3rd: **Growth factor-specific signaling pathway stimulation and gene expression mediated by ErbB receptors**. *J Biol Chem* 2001, **276**(25):22685-22698.
26. Kasper D, Dittmer F, von Figura K, Pohlmann R: **Neither type of mannose 6-phosphate receptor is sufficient for targeting of lysosomal enzymes along intracellular routes**. *J Cell Biol* 1996, **134**(3):615-623.
27. Ludwig T, Ovitt CE, Bauer U, Hollinshead M, Remmler J, Lobel P, Ruther U, Hoflack B: **Targeted disruption of the mouse cation-dependent mannose 6-phosphate receptor results in partial missorting of multiple lysosomal enzymes**. *Embo J* 1993, **12**(13):5225-5235.
28. Stockli J, Rohrer J: **The palmitoyltransferase of the cation-dependent mannose 6-phosphate receptor cycles between the plasma membrane and endosomes**. *Mol Biol Cell* 2004, **15**(6):2617-2626.
29. Asmal M, Colgan J, Naef F, Yu B, Lee Y, Magnasco M, Luban J: **Production of ribosome components in effector CD4+ T cells is accelerated by TCR stimulation and coordinated by ERK-MAPK**. *Immunity* 2003, **19**(4):535-548.
30. De Marcucci O, Lindsay JG: **Component X. An immunologically distinct polypeptide associated with mammalian pyruvate dehydrogenase multi-enzyme complex**. *Eur J Biochem* 1985, **149**(3):641-648.
31. Hiromasa Y, Fujisawa T, Aso Y, Roche TE: **Organization of the cores of the mammalian pyruvate dehydrogenase complex formed by E2 and E2 plus the E3-binding protein and their capacities to bind the E1 and E3 components**. *J Biol Chem* 2004, **279**(8):6921-6933.
32. Kang-Decker N, Mantchev GT, Juneja SC, McNiven MA, van Deursen JM: **Lack of acrosome formation in Hrb-deficient mice**. *Science* 2001, **294**(5546):1531-1533.
33. Su L, Hershberger RJ, Weissman IL: **LYAR, a novel nucleolar protein with zinc finger DNA-binding motifs, is involved in cell growth regulation**. *Genes Dev* 1993, **7**(5):735-748.
34. Bellefroid EJ, Kobbe A, Gruss P, Pieler T, Gurdon JB, Papalopulu N: **Xiro3 encodes a Xenopus homolog of the Drosophila Iroquois genes and functions in neural specification**. *Embo J* 1998, **17**(1):191-203.

35. Briscoe J, Pierani A, Jessell TM, Ericson J: **A homeodomain protein code specifies progenitor cell identity and neuronal fate in the ventral neural tube**. *Cell* 2000, **101**(4):435-445.
36. Christoffels VM, Keijser AG, Houweling AC, Clout DE, Moorman AF: **Patterning the embryonic heart: identification of five mouse Iroquois homeobox genes in the developing heart**. *Dev Biol* 2000, **224**(2):263-274.
37. Kiecker C, Lumsden A: **Hedgehog signaling from the ZLI regulates diencephalic regional identity**. *Nat Neurosci* 2004, **7**(11):1242-1249.
38. Imai K, Imazawa Y, Yao Y, Yamamoto K, Hisatake K, Muramatsu M, Nogi Y: **The fission yeast rpa17+ gene encodes a functional homolog of AC19, a subunit of RNA polymerases I and III of Saccharomyces cerevisiae**. *Mol Gen Genet* 1999, **261**(2):364-373.
39. Yao Y, Yamamoto K, Nishi Y, Nogi Y, Muramatsu M: **Mouse RNA polymerase I 16-kDa subunit able to associate with 40-kDa subunit is a homolog of yeast AC19 subunit of RNA polymerases I and III**. *J Biol Chem* 1996, **271**(51):32881-32885.
40. Kuge O, Nishijima M, Akamatsu Y: **A Chinese hamster cDNA encoding a protein essential for phosphatidylserine synthase I activity**. *J Biol Chem* 1991, **266**(35):24184-24189.
41. Voelker DR, Frazier JL: **Isolation and characterization of a Chinese hamster ovary cell line requiring ethanolamine or phosphatidylserine for growth and exhibiting defective phosphatidylserine synthase activity**. *J Biol Chem* 1986, **261**(3):1002-1008.
42. Krahn AK, Ma K, Hou S, Duronio V, Marshall AJ: **Two distinct waves of membrane-proximal B cell antigen receptor signaling differentially regulated by Src homology 2-containing inositol polyphosphate 5-phosphatase**. *J Immunol* 2004, **172**(1):331-339.
43. Marshall AJ, Krahn AK, Ma K, Duronio V, Hou S: **TAPP1 and TAPP2 are targets of phosphatidylinositol 3-kinase signaling in B cells: sustained plasma membrane recruitment triggered by the B-cell antigen receptor**. *Mol Cell Biol* 2002, **22**(15):5479-5491.
44. Okkenhaug K, Vanhaesebroeck B: **PI3K in lymphocyte development, differentiation and activation**. *Nat Rev Immunol* 2003, **3**(4):317-330.
45. Behrens A, Sibilia M, Wagner EF: **Amino-terminal phosphorylation of c-Jun regulates stress-induced apoptosis and cellular proliferation**. *Nat Genet* 1999, **21**(3):326-329.
46. Bohmann D, Ellis MC, Staszewski LM, Mlodzik M: **Drosophila Jun mediates Ras-dependent photoreceptor determination**. *Cell* 1994, **78**(6):973-986.
47. Bossy-Wetzel E, Bakiri L, Yaniv M: **Induction of apoptosis by the transcription factor c-Jun**. *Embo J* 1997, **16**(7):1695-1709.
48. Chen J, Stewart V, Spyrou G, Hilberg F, Wagner EF, Alt FW: **Generation of normal T and B lymphocytes by c-jun deficient embryonic stem cells**. *Immunity* 1994, **1**(1):65-72.
49. Eferl R, Wagner EF: **AP-1: a double-edged sword in tumorigenesis**. *Nat Rev Cancer* 2003, **3**(11):859-868.
50. Ham J, Babij C, Whitfield J, Pfarr CM, Lallemand D, Yaniv M, Rubin LL: **A c-Jun dominant negative mutant protects sympathetic neurons against programmed cell death**. *Neuron* 1995, **14**(5):927-939.
51. Lord KA, Abdollahi A, Hoffman-Liebermann B, Liebermann DA: **Proto-oncogenes of the fos/jun family of transcription factors are positive regulators of myeloid differentiation**. *Mol Cell Biol* 1993, **13**(2):841-851.

52. Schreiber M, Kolbus A, Piu F, Szabowski A, Mohle-Steinlein U, Tian J, Karin M, Angel P, Wagner EF: **Control of cell cycle progression by c-Jun is p53 dependent**. *Genes Dev* 1999, **13**(5):607-619.
53. Miyaji M, Jin ZX, Yamaoka S, Amakawa R, Fukuhara S, Sato SB, Kobayashi T, Domae N, Mimori T, Bloom ET *et al*: **Role of membrane sphingomyelin and ceramide in platform formation for Fas-mediated apoptosis**. *J Exp Med* 2005, **202**(2):249-259.
