# Supplementary material for: A Myc-regulated transcriptional network controls B-cell fate in response to BCR triggering
Source: BMC Genomics. 2009 Jul 17;10:323. doi: 10.1186/1471-2164-10-323 (PMC2722676; doi:10.1186/1471-2164-10-323)
Supplement: Additional file 5 — Statistical analysis of cis-acting sequences in promoter regions of co-regulated genes. (A) Co_clustering of the five focus gene, Ptger4, Marcksl1, Myc, Crsp9 and Ifrd1, by k-means analysis is independent of the selected k value. Each number in the table presents the identification number of a cluster. Note that the data from two different probes each (a, b) for Marcksl1 and Myc were used for clustering. (B) The significance of the predicted Pax5 and ETS family members' binding sites in the promoter regions of was evaluated using the Genomatix software (see also Figure 2B). [file 1471-2164-10-323-S5.pdf]

A

**Number of clusters (k)**	***Ptger4***	***Marcksl1*[(a)]**	***Marcksl1*[(b)]**	***Myc*[(a)]**	***Myc*[(b)]**	***Crsp9***	***Ifrd1***
**8**	4	4	4	4	4	4	4
**9**	9	9	9	9	9	9	9
**10**	5	5	5	5	5	5	5
**11**	2	2	2	2	2	2	2
**12**	11	11	11	11	11	11	11
**13**	13	13	13	13	13	13	13
**14**	8	8	8	8	8	8	8
**15**	14	14	14	14	14	14	14
**16**	14	14	14	14	14	14	14
**17**	2	2	2	2	2	2	2
**18**	2	2	2	2	2	2	2
**19**	13	13	13	13	13	13	13
**20**	9	9	9	9	9	9	9

B

**TF**	**Number of sequences**	**Number of matches**	**Expected (genome)**	**Overrepresentation (genome)**	**Z-Score (genome)**	**Expected (promoters)**	**Overrepresentation (promoters)**	**Z-Score (promoters)**
Pax5	6	21	7.1	2.96	5.03	13.8	1.52	1.8
ETS family	6	65	53.05	1.23	1.57	58.71	1.11	0.76
